# A Novel Multimodal Radiomics Model for Preoperative Prediction of Lymphovascular Invasion in Rectal Cancer

**DOI:** 10.3389/fonc.2020.00457

**Published:** 2020-04-07

**Authors:** Yiying Zhang, Kan He, Yan Guo, Xiangchun Liu, Qi Yang, Chunyu Zhang, Yunming Xie, Shengnan Mu, Yu Guo, Yu Fu, Huimao Zhang

**Affiliations:** ^1^Department of Radiology, The First Hospital of Jilin University, Changchun, China; ^2^GE Healthcare, Shanghai, China

**Keywords:** lymphovascular invasion, rectal cancer, multimodal imaging, computed tomography, MRI, radiomics, nomogram

## Abstract

**Objective:** To explore a new predictive model of lymphatic vascular infiltration (LVI) in rectal cancer based on magnetic resonance (MR) and computed tomography (CT).

**Methods:** A retrospective study was conducted on 94 patients with histologically confirmed rectal cancer, they were randomly divided into training cohort (*n* = 65) and validation cohort (*n* = 29). All patients underwent MR and CT examination within 2 weeks before treatment. On each slice of the tumor, we delineated the volume of interest on T2-weighted imaging, diffusion weighted imaging, and enhanced CT images, respectively. A total of 1,188 radiological features were extracted from each patient. Then, we used the student *t*-test or Mann–Whitney *U*-test, Spearman's rank correlation and least absolute shrinkage and selection operator (LASSO) algorithm to select the strongest features to establish a single and multimodal logic model for predicting LVI. Receiver operating characteristic (ROC) curves and calibration curves were plotted to determine how well they explored LVI prediction performance in the training and validation cohorts.

**Results:** An optimal multi-mode radiology nomogram for LVI estimation was established, which had significant predictive power in training (AUC, 0.884; 95% CI, 0.803–0.964) and validation (AUC, 0.876; 95% CI, 0.721–1.000). Calibration curve and decision curve analysis showed that the multimodal radiomics model provides greater clinical benefits.

**Conclusion:** Multimodal (MR/CT) radiomics models can serve as an effective visual prognostic tool for predicting LVI in rectal cancer. It demonstrated great potential of preoperative prediction to improve treatment decisions.

## Introduction

Colorectal cancer is the third most common cancer in the world, and by 2030 there will be about 2.2 million cases worldwide (1). Lymphovascular invasion (LVI) is defined as the presence of tumor cells within the endothelia-lined luminal space or the destruction of the lymphovascular wall by tumor cells (2). The dissemination of cancer cells through lymphatic channels or venules may be a crucial step in the early stages of lymph node metastasis (3). LVI, which is associated with poor prognosis and is a high-risk factor for recurrence after endoscopic surgery, has been recognized as an important prognostic determinant that is independent of stage in colorectal cancer (4–6). The National Comprehensive Cancer Network Clinical Practice Guidelines recommend preoperative chemo radiotherapy for patients with T3N0M0 disease (7), which may also be necessary in the presence of LVI (8). Although MRI is a reproducible and accurate method for preoperatively identifying vessels larger than 3 mm, due to its moderate sensitivity, imaging assessment of LVI is difficult and smaller vein invasion may be overlooked (9, 10). Also, preoperative biopsy assessment, depending on tumor size, may suffer from a considerable sampling error, thus contributing to an unknown rate of missed diagnoses. Hence, the evaluation of new imaging biomarkers for predicting LVI preoperatively may contribute to improved patient care.

Multimodal machine learning (MMML) aims to attain the ability of processing and understanding multimodal information by machine learning. Multimodal fusion is used for combining information of multiple modalities and performing target prediction (classification or regression) (11–13). Medical imaging contains data in different modalities such as CT, MRI, PET, ultrasound, and X-ray. T2WI and DWI are accepted as routine examinations for defining the locoregional clinical stage of rectal cancer (14, 15). DWI can better reflect the volume of tumor and distinguish fibrosis. Compared with other single phase imaging, the portal venous phase of contrast enhanced CT (CE-CT) protocol performs satisfactory preoperative evaluation of TNM staging in patients with colorectal cancer preoperatively (16). Although there have been advances in new medical imaging technologies, highly trained experts are still required to interpret these modalities for diagnosis (12). Multimodal fusion techniques can be divided into pixel, feature, and decision levels, which are used to fuse original data and abstract features and decision results, respectively (11–13).

Meanwhile, radiomics is a new method of medical image analysis that further characterizes the phenotype of tumor by transforming conventional medical images into quantitative, high-dimensional, and exploitable radiology data (17–20). Currently, many studies have applied radiomics characteristics to predict lymph node metastasis and perineural invasion (PNI) in colorectal cancer (21, 22), evaluate the response to neoadjuvant therapy (23), determine preoperative synchronous distant metastasis (24), and predict staging of rectal cancer (25). However, its use in LVI prediction is still rare.

In this study, we combined two new imaging technologies and explored the advantage of a multimodal radiomics model from MR and CT images for individualized preoperative prediction of LVI in rectal cancer.

## Materials and Methods

### Patients

**Figure 1:**
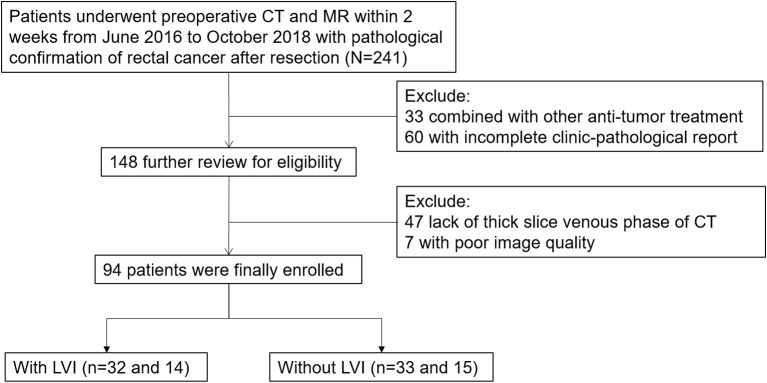
Flow diagram of patient selection.

The study was approved by the Ethics Committee of the First Hospital of Jilin University. From June 2016 to October 2018, 241 primary rectal cancer patients were enrolled based on the following inclusion standard: (1) patients diagnosed with pathologically proven rectal cancer as documented in the medical records of our institution and (2) had received preoperative CT and MR within 2 weeks, as documented in PACS and medical records. The exclusion criteria included: (1) anti-tumor treatments (*n* = 33); (2) incomplete clinic-pathological reports (*n* = 60); (3) lack of thick slices of venous phase CT (*n* = 47); (4) poor image quality (*n* = 7). Finally, the study population consisted of 94 patients. Figure 1 demonstrates a flow diagram of patient selection. The entire cohort was divided into a training cohort (*n* = 65) and validation cohort (*n* = 29) randomly at a 7:3 ratio. The training cohort was used to build single and multimodal radiomics models that were evaluated by the validation cohort.

### Image Acquisition

All MR images were acquired on a 3.0-T MRI scanner (Ingenia, Philips Medical Systems, Netherlands). The scan parameters were as follows: High-resolution axial T2WI was performed using fast recovery fast spin echo, repetition time (TR) = 3,500 ms, echo time (TE) = 100 ms, slice thickness = 3.0 mm, gap = 0.3 mm, matrix = 288 × 256, echo train length = 24, and field of view (FOV) = 18 × 18 cm; DWI was performed with b = 1,000 s/mm^2^, TR = 2,800 ms, TE =70 ms, slice thickness = 4.0 mm, matrix = 256 × 256, FOV = 34 × 34 cm, and gap = 1.0 mm.

**Figure 2:**
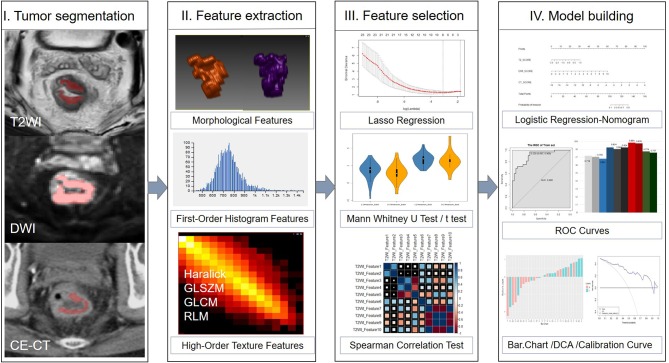
Framework of this study. A flowchart describing the radiomics method for LVI of rectal cancer prediction.

CE-CT was performed either on a 256-detector row (Brilliance, Philips Medical Systems, Netherlands) or on a 64 slice dual source CT scanner (Definition, Siemens Medical Systems, Germany). Both machines' protocol parameters were the same: tube current = 250 mA; tube voltage = 120 kV; slice thickness = 5 mm. 1.5 mL/kg of iodinated contrast media was injected into vein via a pump injector at a rate of 2.0−3.0 mL/s after a routine unenhanced scan. Venous phase CT images were obtained at 60 s. Figure 2 depicts a flowchart of this study.

### Tumor Segmentation

Tumor segmentation was conducted by using an open source software package (ITK-SNAP, version 3.4.0, www.itksnap.org). Two independent radiologists (reader 1 with 3 years of experience in abdominal imaging, and reader 2 with 5 years) outlined the volumes of interest (VOIs) manually which around the lesion avoiding normal signal regions from T2WI, DWI, and CE-CT images on each tumor slice, respectively. Neither radiologist was aware of the clinicopathological results or the imaging interpretations of other readers.

The VOI was defined as follows: (1) the whole primary tumor on T2WI was defined by outlining the contour of the tumor on slightly high signal; (2) the VOI on DWI (b = 1,000 s/mm^2^) was covered on the high signal intensity region; and (3) on CE-CT imaging, the whole primary tumor was drawn along the abnormal region which enhanced heterogeneously in the venous phase. All VOIs were segmented on each slice manually, which contained the chords and burrs surrounding lesions and excluded the fluid in the intestinal lumen. VOI in each sequence showed the tumor segmentation in Figure 2.

### Radiomics Features Extraction

On each modality, 396 radiomics features were extracted from the VOIs using the A.K. software (Artificial Intelligence Kit, AK, version V3.0.0.R, GE Healthcare, China), including 42 first order histogram features, 9 morphological features, 10 Haralick features, 11 gray-level zone size matrix (GLZSM), 144 gray-level co-occurrence matrix (GLCM) with an offset of 1/4/7, and 180 gray-level run-length matrix (RLM) with an offset of 1/4/7. Finally, 1,188 radiomics features were generated from each patient. Detailed information about the extracted features is provided in Supplementary Figure 1.

We used intra- and inter-class correlation coefficients (ICCs) to assess the intra-observer and inter-observer reproducibility of feature extraction. We initially chose 30 VOIs from each modality randomly. The intra-observer ICC was calculated by comparing reader 2 twice segmentation (repeated at 7-day intervals). The inter-observer ICC was calculated by comparing the extraction of reader 1 and those of reader 2 (first time). When the ICC exceeded 0.75, it was considered as good agreement.

### Feature Selection and Model Building

Features selection and model building were performed on RStudio-1.1.463. To explore the strongest features which were correlated best with histopathology, we used student *t*-test or Mann–Whitney *U*-test, and least absolute shrinkage selection operator (LASSO) regression to reduce over-fitting or selection bias in our radiomics model. To reduce the redundancy of the features, Spearman's rank correlation was used to remove features with high correlation (here we chose coefficient |r| ≥ 0.9). The radiomics score, which included the CT_Score, DWI_Score, and T2_Score, respectively, were generated by using the selected features according to the linear combination weighted by their respective coefficients from each patient. The multimodal radiomics was constructed by two different methods: Model A, which named Rad-score_A, was based on CT_Score, DWI_Score, and T2_Score. Model B, which named Rad-score_B, was based on 396^*^3 radiomics features. The details of LASSO regression are shown in the Supplementary. Delong tests were used to compare the differences between the two multimodal radiomics models, and the better model was chosen to generate the radiomics nomogram.

### Validation and Nomogram Construction

Accuracy, specificity, sensitivity, and the area under the receiver operating characteristic curve (AUC) were used to estimate the predictive performance of the radiomics models. The calibration curve depicted the performance characteristics of the multimodal radiomics models graphically. A radiomics nomogram from the best model was constructed depending on the multivariate logistic regression model. We use the Hosmer-Lemeshow test to assess the goodness-of-fit of the nomogram and use a decision curve analysis to evaluate the clinical usefulness of the multimodal radiomics via calculating the net benefit at different threshold probabilities. Moreover, we carried out stratification analysis based on different CT protocols.

### Statistical Analysis

All statistical analyses were performed with RStudio Server (Version 1.1.463; RStudio, Inc, Boston, MA, USA). The student *t-*test and Mann–Whitney *U*-tests were performed when appropriate to compare continuous variables. A chi-squared test was used for classified variables between groups. The radiomics scores were expressed as median (25 quantile, 75 quantile), and the continuous variables were expressed as mean ± standard deviation (SD). Intra- and inter-class correlation coefficients (ICC) were performed to evaluate the effects of variations between intra- and inter-readers in the extracted radiomics features. All statistical tests were two-sided, and *P*-values of <0.05 presented statistically significant statistical analysis. LASSO regression analysis was performed using the “glmnet” package. Multivariate logistic regression, nomogram and calibration curves were generated using the “rms” package. ROC curves were plotted with the “pROC” package. Decision curve analysis was done using the function of “dca. R.”

## Results

### Patients Characteristics

Table 1 shows the clinical characteristics of the patients. Our study included 46 (48.9%) patients with LVI and 48 (51.1%) patients without LVI. Clinical characteristics of LVI-positive and LVI-negative groups were not statistically different in training and validation cohorts (*p* = 0.329–0.718), except for the Rad-score (*p* < 0.05). The pathological staging (T, N stage) of LVI-positive and LVI-negative groups were statistically different in training and validation cohorts. However, our model was established preoperatively, the relevant postoperative clinical characteristics were not included in our model.

**Table 1:** Clinical characteristics and radiomics score of the training and validation cohort for lymphovascular invasion of rectal cancer.

**Variables**		**Training (*****n*** **=** **65)**			**Validation (*****n*** **=** **29)**		
		**LVI(+) (*n* = 32)**	**LVI(–) (*n* = 33)**	***P*-value**	**LVI(+) (*n* = 14)**	**LVI(–) (*n* = 15)**	***P*-value**
Age (Mean ± SD)		59.56 ± 10.96	60.61 ± 13.18	0.541	55.57 ± 14.12	58.73 ± 12.62	0.718
Gender (No., %)	Male	22 (68.75)	25 (75.76)	0.535	12 (85.71)	12 (80.00)	0.697
	Female	10 (31.25)	8 (24.24)		2 (15.29)	3 (20.00)	
CEA (ng/ml, %)	Normal	19 (59.38)	16 (48.48)	0.386	6 (42.86)	8 (53.33)	0.589
	Abnormal	13 (40.62)	17 (51.52)		8 (57.14)	7 (46.67)	
CA-199 (ng/ml, %)	Normal	24 (75.00)	28 (84.85)	0.329	12 (85.71)	12 (80.00)	0.697
	Abnormal	8 (25.00)	5 (15.15)		2 (14.29)	3 (20.00)	
CT_Score [Median (25, 75%)]		1.222 (−0.158, 2.918)	−0.911 (−2.456, 0.599)	<0.001^*^	1.530 (0.835, 2.170)	−1.684 (−4.073, −0.145)	0.002^*^
DWI_Score [Median (25, 75%)]		0.468 (−0.005, 1.259)	0.070 (−0.593, 0.500)	0.012^*^	0.232 (−0.037, 0.509)	−0.354 (−1.826, −0.176)	0.002^*^
T2_Score [Median (25, 75%)]		0.331 (−0.057, 0.724)	−0.268 (−1.106, 0.184)	0.002^*^	0.528 (0.193, 0.808)	−0.374 (−0.780, 0.223)	0.063
Rad-score A [Median (25, 75%)]		1.678 (0.232, 2.380)	−1.411 (−2.391, −0.628)	<0.001^*^	0.657 (0.583, 1.565)	−2.510 (−3.41, −1.38)	<0.001^*^
Rad-score B [Median (25, 75%)]		0.529 (−0.215, 0.753)	−0.485 (−1.096, 0.035)	<0.001^*^	0.488 (−0.088, 0.780)	−0.291 (−1.164, 0.277)	0.018^*^
pT stage	T1-2	3 (9.38)	19 (57.58)	<0.001^*^	1 (7.14)	0 (0.00)	0.168
	T3-4	29 (90.62)	14 (42.42)		13 (92.86)	15 (100.00)	
pN stage	N0	0 (0.00)	32 (96.97)	<0.001^*^	0 (0.00)	14 (93.33)	<0.001^*^
	N1-2	32 (100.00)	1 (3.03)		14 (100.00)	1 (6.67)	
Tumor length (Mean ± SD)		5.57 ± 2.36	5.89 ± 2.26	0.447	5.32 ± 1.59	5.32 ± 1.95	0.856
Tumor thickness (Mean ± SD)		1.56 ± 0.79	1.50 ± 0.78	0.419	1.30 ± 0.37	1.45 ± 0.55	0.493
T2WI_volume (mm^3^) [Median (25, 75%)]		7558.770 (4257.333, 11021.175)	5934.650 (3059.595, 11771.650)	0.550	6097.545 (2727.905, 7122.2125)	7244.400 (3876.030, 10126.800)	0.346
DWI_volume (mm^3^) [Median (25, 75%)]		7492.685 (4209.595, 9908.308)	6503.910 (2510.985, 13763.400)	0.659	4833.990 (3279.730, 7781.370)	7009.280 (4028.330, 10527.300)	0.167
CT_volume (mm^3^) [Median (25, 75%)]		9748.935 (6104.673, 5280.300)	12227.700 (4606.160, 16144.450)	0.959	8004.380 (3894.278, 10866.125)	11366.800 (7116.990, 17447.800)	0.181

*LVI, lymphovascular invasion; CEA, Carcinoembryonic antigen; CA-199, Carbohydrate antigen 199; Rad-score, Radiomic score; pT stage, pathological tumor stage; pN stage, pathological limph node stage*.

* *p < 0.05 indicated significant differences*.

The intra-observer reproducibility of feature extraction based on twice-extracted features of reader 2 was satisfactory. Therefore, the remaining image segmentation was performed by reader 2. After analysis of reproducibility to avoid the effect of intra/inter observer variation, 110 radiomic features on tumor regions remained for T2WI; 146 tumor features for DWI; and 102 tumor features for CE-CT. We used volume MM, which is volume (unite: mm^3^) of the VOI, in the radiomics feature that belongs to the form factor features, to represent tumor size. The smallest tumor included in the study was 246.533 mm^3^on T2WI, which is 187 voxels in size (voxel size: 0.625 × 0.703 × 3 mm^3^), with maximal dimensions of 0.4 cm in the axial level, and 2.6 cm in the sagittal plane.

### Single Radiomics Model and Evaluation

**Figure 3:**
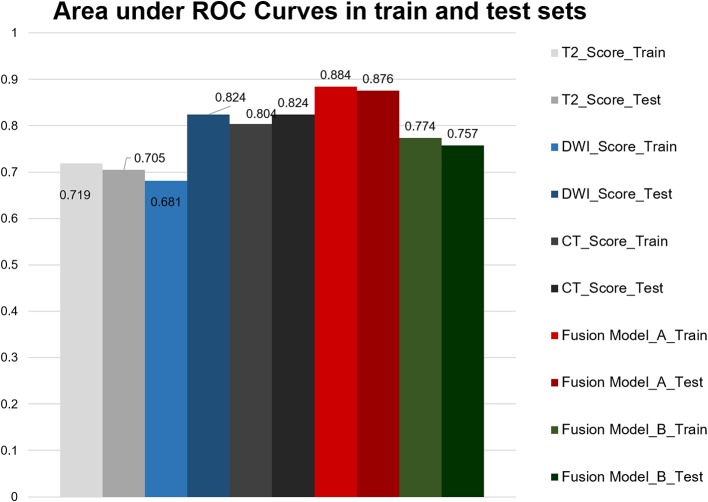
Area under the ROC curves of each radiomics model.

The selected features for single radiomics signatures were calculated from each modality. Single radiomics signatures from T2WI, DWI, and CE-CT were verified with AUC (Figure 3). The radiomics signature by single modality was established with a Rad score calculated as follows:

CT_score = −3.62+(−4.75 × 10^−14^ × ClusterProminence_AllDirection_offset1_SD

−5.40 × 10^−5^ × ClusterShade_angle45_offset7 −6.45 × 10^3^ × Correlation_angle135_offset7
+2.85 × 10^−9^ × HaralickCorrelation_angle90_offset7 + 1.53 × 10^−3^ × Inertia_angle45_offset7
−7.41 × 10 × InverseDifferenceMoment_angle45_offset7
−1.49 × 10^5^× ShortRunEmphasis_AllDirection_offset7_SD
+5.60 × SmallAreaEmphasis)

T2WI_score = −77.9 + (−5.92 × 10^−3^

× HighGreyLevelRunEmphasis_AllDirection_offset4_SD
+8.01 × 10^−4^ × HighGreyLevelRunEmphasis_AllDirection_offset7_SD
+2.35 × 10^−1^ × LongRunEmphasis_angle45_offset7
+8.12 × 10× ShortRunEmphasis_angle45_offset4
+1.20 ×10^−4^ ×ShortRunHighGreyLevelEmphasis_AllDirection_offset7_SD
−4.20 × Sphericity)

DWI_score = −213.1 + (21.8 × GLCMEnergy_angle45_offset7

+122.4 × ShortRunEmphasis_angle135_offset4
+ 92.1 × ShortRunEmphasis_angle45_offset4).

### Multimodal Radiomics Model and Evaluation

The Rad-score A was constructed from integration formulas for single radiomics signatures derived from each modality (T2WI, DWI and CE-CT) using the formula:

*The Rad-score A* = −0.360 + (−0.897 ×T2WI_score +0.6267 × DWI_score+0.609× CT_score)

The Rad-score B was three radiomics features with non-zero coefficients (one feature from T2-weighted images and two features from CE-CT images) selected from a total 1,188 features simultaneously using the formula:

*The Rad-score B* = 73.55 + (−7.48 × T2WI_Sphericity-3854.49 × CT_Correlation_angle135_offset7 + 78.75 × CT_ShortRunEmphasis_angle135_offset4)

The optimum cut-off value of each radiomics models as per the Youden index, as shown in Table 2. Patients were divided into either positive or negative predictions for LVI based on Rad-scores.

**Table 2:** The performance of each models in the train and validation cohorts.

**Model**	**AUC (95% CI lower-upper)**		**Specificity**		**Sensitivity**		**Accuracy**		**Cutoff**	***P*-value**
	**Train**	**Validation**	**Train**	**Validation**	**Train**	**Validation**	**Train**	**Validation**		
CT	0.804 (0.697–0.911)	0.824 (0.643–1.000)	0.545	0.600	0.969	0.857	0.754	0.724	0.336	0.854
DWI	0.681 (0.551–0.811)	0.824 (0.667–0.981)	0.939	1.000	0.375	0.143	0.662	0.586	0.594	0.174
T2WI	0.719 (0.591–0.846)	0.705 (0.501–0.909)	0.697	0.600	0.750	0.786	0.723	0.690	0.502	0.910
Model A	0.884 (0.803–0.964)	0.876 (0.721–1.000)	0.727	0.800	0.938	0.929	0.831	0.862	0.313	0.935
Model B	0.774 (0.654–0.894)	0.757 (0.576–0.938)	0.788	0.533	0.781	0.786	0.785	0.655	0.477	0.942

*P was derived from Delong test between the train and validation cohorts*.

### Performance Comparison and Calibration

We tried our best to build a stable model, thus 10-fold cross validation was repeated 50 times in the training cohort when applying the LASSO method so as to generate the optimal lambda that can construct a robust model in the logistic regression analysis. Meanwhile, delong test was used to explore if the model was robust not only in the training cohort but also in the validation cohort. Delong test *P*-value exceeded a value of 0.05 when comparing the performance of the model in training and validation cohort.

We measured AUC values of each model including single model, model A and model B (Figure 3). The AUC of the CT model in the training cohort was 0.804 (95% CI, 0.697–0.911) and in the validation cohort was 0.824 (95% CI, 0.643–1.000), with no statistically significant difference between the two cohorts (*P* = 0.854). The T2WI model yielded an AUC of 0.719 (95% CI, 0.591–0.846) and 0.705 (95% CI, 0.501–0.909), also with no significant difference between each cohort (*P* = 0.910). The AUC of the training cohort of DWI model was 0.681 (95% CI, 0.551–0.811), and the AUC of the validation cohort was 0.824 (95% CI, 0.667–0.981), with no significant difference between groups (*P* = 0.174). Accuracy, sensitivity, specificity, and AUC of each radiomics model are shown in Table 2. And the box plots are shown in Supplementary Figure 2.

**Figure 4:**
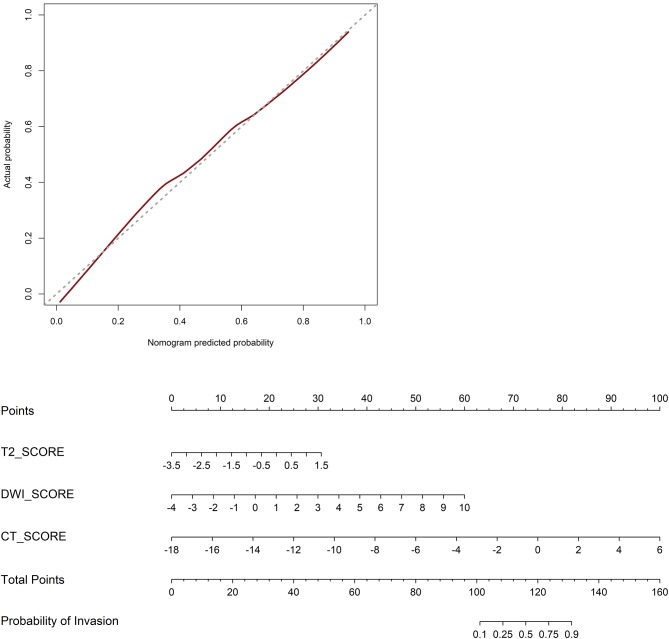
Calibration curves of the nomogram in the validation cohort. The closer fit of the diagonal curved line to the ideal straight line indicates the predictive accuracy of the nomogram from the best model. Radiomics nomogram was developed in the training cohort.

**Figure 5:**
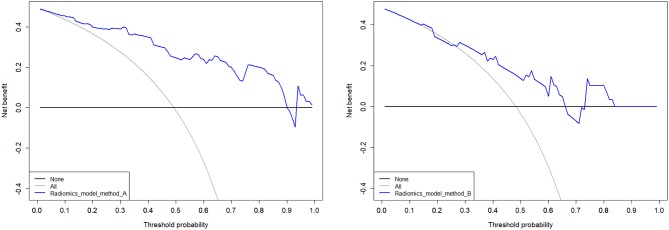
Decision curves analysis of multimodal A and B performed in the validation cohort. The net benefit is represented on the y-axis. The threshold probability is represented on the x-axis. The net benefit of model A was higher than model B across the full range of reasonable threshold probabilities.

Model A achieved the highest AUC of all models (AUC = 0.884 and 0.876, respectively), with a high sensitivity and specificity in the training and validation cohorts (sensitivity = 0.938 and 0.929, specificity = 0.727 and 0.800). The AUC of model B was 0.774 in the training cohort and 0.757 in the validation cohort, with sensitivity and specificity in the training and validation cohorts (sensitivity = 0.781 and 0.786, specificity = 0.788 and 0.533). The calibration curves depicted that the predicted risks were consistent with the observed outcomes of LVI. The closer fit of the diagonal curved line to the ideal straight line indicates the predictive accuracy of the nomogram from the best model (Figure 4). The DCA indicated that model A was the best method across the full range of reasonable threshold probabilities (Figure 5). The stratified analysis showed that the performance of model A was not affected by the CT version (Supplementary Figure 3). The AUC of models derived from images acquired on the PHILIPS scanner was 0.880 (95% CI, 0.797–0.963) and the AUC of models derived from images acquired on the Siemens scanner was 0.833 (95% CI, 0.631–1.00, Delong test *P* > 0.05).

### Discussion

To the best of our knowledge, this is the first study to develop a multimodal radiomics model, using radiomics features from MR and CT to predict LVI in rectal cancer. The challenges of multimodal fusion mainly include how to judge the confidence level of each modality and the correlation between modalities, how to reduce the dimension of multimodal characteristic information, and how to register the multimodal data collected asynchronously (11–13). We compared the advantages of two multimodal radiomics models for CT and MRI integration. The model A was superior to the single model and the model B, indicating that the multimodal radiomics model approach may have a greater value in preoperative LVI prediction. The multimodal model can provide more abundant information than either modality alone.

In a prior study, Kim et al. (10) reported an excellent specificity but limited sensitivity of 93.2 and 68.2% of MRI for detecting LVI in rectal cancer. Later, Chen et al. (26) performed DWI- and T2-weighted MRI–based gross tumor volume (GTV) to measure LVI in 50 patients. The sensitivity of DWI-based GTV was similar to that of T2-weighted MRI–based GTV (91.7 vs. 91.7%), whereas the specificity of DWI-based GTV was higher than T2-weighted MRI–based GTV (82.6 vs. 79.3%). Their results are similar to our study, but in our final model, the volume feature was not retained after feature selection, possibly because volume feature is biased by subjective assessment of the observer. Jiang et al. (27) combined clinical factor and radiomic score to build a model for predicting pathological stage. The aim of our study, however, was to investigate the influence of multimodal fusion on radiomics models using different methods, and therefore clinical factors had not been included into the model. Moreover, as shown in Table 1, clinical characteristics were not statistically different in training and validation cohorts.

There have been no studies on lymphovascular invasion of rectal cancer using radiomics. Lymphovascular invasion has been studied in breast (28); colorectal (29); and endometrial cancers (30); and urinary tract urothelial carcinoma (31). However, radiomics was only used in the lymphovascular invasion of breast cancer (32). Radiomics mainly improves the prediction performance of medical images by improving medical image analysis and using computer algorithms to extract thousands of quantitative features (19). Although these characteristics can reflect tumor biology behavior from various aspects (33), the correlation is also difficult to comprehend between single radiomics features and biological behaviors. Moreover, constructing multi-feature panels is a more common evaluation method (34, 35). We integrated the remaining features of feature selection into a single radiomics score to reflect information more effectively.

We considered that the performance of the multimodal radiomics model was deeply influenced by the process of the feature selection, although we used 10-fold cross validation. In method B, a total of 1,188 features were mixed for the feature selection, in which the features derived from CT images, DWI, and T2WI were treated equally without discrimination. Thus, there was an inevitable bias, which could be eliminated by using a larger patient data base including thousands of cases in a big-data-approach. Meanwhile, we established another fusion model using method A, which combined the radiomics scores calculated based on CT image, DWI, and T2WI, respectively. Model A is superior to B because the model B can only judge the features from different modalities separately, ignoring the correlation between modalities, and excluding those with small contribution value. Hence, model B only includes the features from T2WI and CT, without DWI. As model A preserves the important information of each model, it should be superior to model B and indeed performed better than model B and visual assessment alone. This could indicate a clinically relevant potential of the proposed radiomics nomogram for preoperative assessment and image guided therapy. Interestingly, features such as Short Run Emphasis and Small Area Emphasis, which could be attributed to second-order texture features, seem to predict vascular invasion more reliably.

Multimodal fusion techniques include pixel, feature, and decision levels (11–13). In our study, we didn't choose pixel but feature level because the two medical examinations could not be performed simultaneously during routine diagnostic procedures. Also, feature level based multimodal fusion offers a very flexible and convenient way to utilize multimodal information. Due to the inevitable gastrointestinal motion, even for simultaneous multimodal imaging, accuracy in image registration for the gastrointestinal tract still poses challenges even for the most sophisticated, existing hybrid imaging modalities. The approach presented in our study, in contrast, is not prone to misregistration errors and can be even more generalized to combine information of different modalities. In contrast to pixel level fusion, feature level fusion using radiomic features simplifies comprehensive integration of multimodal information which, given its numerical derivation, could allow to include any other data source such as ultrasound data, lab test results or genomic data.

By comparing AUC values, the AUC of T2WI and CT are higher. However, combining DWI makes the model more efficient but cannot provide more texture features. Hence DWI has critical but limited value. Moreover, the calibration curve of the predictive model demonstrated good agreement between the predictive and actual probabilities. In our study, the calibration curve depicted that the model A better predicted actual LVI in rectal cancer in the validation cohort than other models. DCA showed that the model A adds more benefit to predicting LVI than model B at any given threshold probability. It seems to be obvious that assessing tumorous disease with single modal radiomics information will not be comprehensive. However, development of methods and strategies for the integration of information of different dimensions is still in its early stages, and combining prediction models, as performed in the current study, might increase their precision and could be extended to other diagnostic indicators. Further research following this scheme is warranted.

This study has several limitations: First, our research was conducted at a single institution. Although all MR images were obtained from a uniform MRI scanner with a standardized imaging acquisition sequence to reduce the bias and variance of our results. Further confirmation from other agencies is needed to improve the robustness of the model. Second, our images were not normalized preprocessing. Currently, there is still no universal criterion for the standardization of imaging, and the results will be influenced by factors including equipment, parameters, and radiomics research methods and so on. We selected images from the same MRI equipment as a normalization strategy. About 78% of CT images were from the same CT version (Brilliance, Philips Medical Systems). The CT voltage value was the same, which is the only factor affecting the radiomics texture characteristics (36). Moreover, the thickness/gap of slices are similar to avoid image preprocessing. The stratified analysis showed that the performance by the different versions of CT was also acceptable. Third, this study is a retrospective study with inevitable deviation. If containing external validation would be better. A prospective study is also needed for further verification. Finally, our study is a single center study, mainly including the patients who performed with CT and MR, so the sample size is small. We will cooperate with other hospitals to explore the robustness of multimodal model in future.

## Conclusions

In conclusion, this study presents a multimodal radiomics model with good discriminative ability in predicting preoperative LVI in rectal cancer. The multimodal model A based on modalities is better than the model B based on features. Such preoperative models of LVI may potentially be useful to modified individualized and accurate treatment strategies.

## Data Availability Statement

The datasets generated for this study are available on request to the corresponding author.

## Ethics Statement

The studies involving human participants were reviewed and approved by First Hospital of Jilin University. Written informed consent for participation was not required for this study in accordance with the national legislation and the institutional requirements.

## Author Contributions

YZ, YaG, YF, and HZ contributed conception and design. KH, XL, YuG, YF, and HZ organized the database. KH, QY, CZ, YX, SM, and HZ administrate, managed patients, and provide technical support, etc. YZ wrote the first draft of the manuscript. YZ and YaG performed the statistical analysis. YaG, XL, YF, and HZ review and revision of the manuscript.

### Conflict of Interest

The authors declare that the research was conducted in the absence of any commercial or financial relationships that could be construed as a potential conflict of interest.
