# Supplementary material for: A Novel Multimodal Radiomics Model for Preoperative Prediction of Lymphovascular Invasion in Rectal Cancer
Source: Front Oncol. 2020 Apr 7;10:457. doi: 10.3389/fonc.2020.00457 (PMC7160379; doi:10.3389/fonc.2020.00457)
Supplement: Supplementary file 1 [file Presentation_1.docx]

**Supplementary Figure1.** Detailed information about the extracted radiomics features.

**
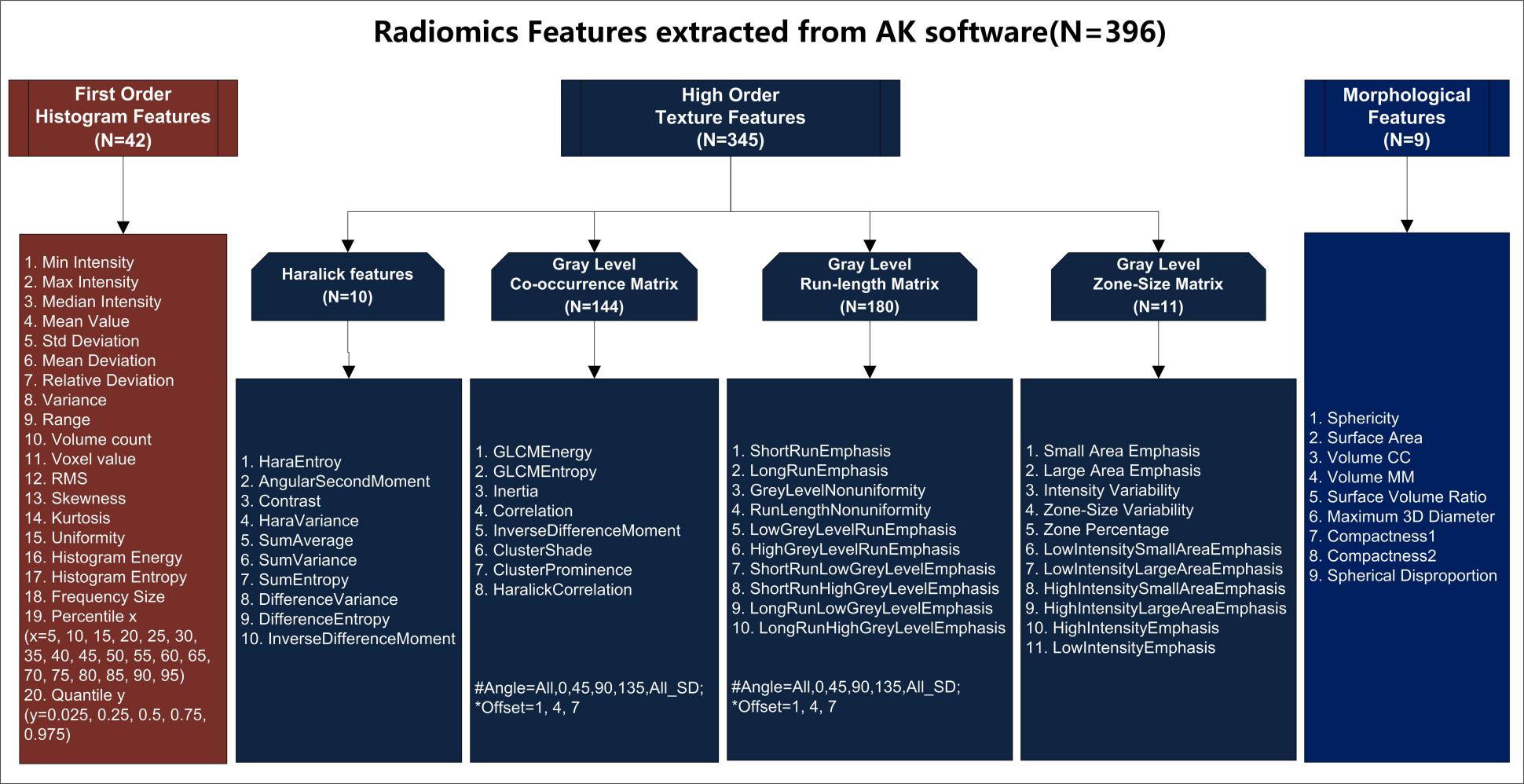
**

**Supplementary Figure2**.The boxplots of each models in the training cohort and the validation cohort with LVI or non-LVI


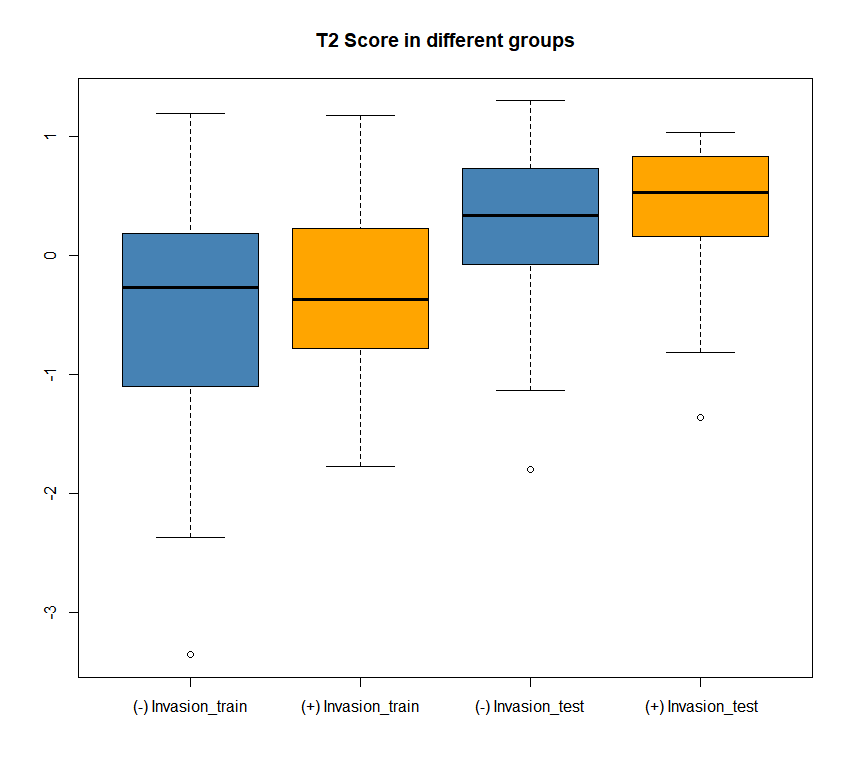

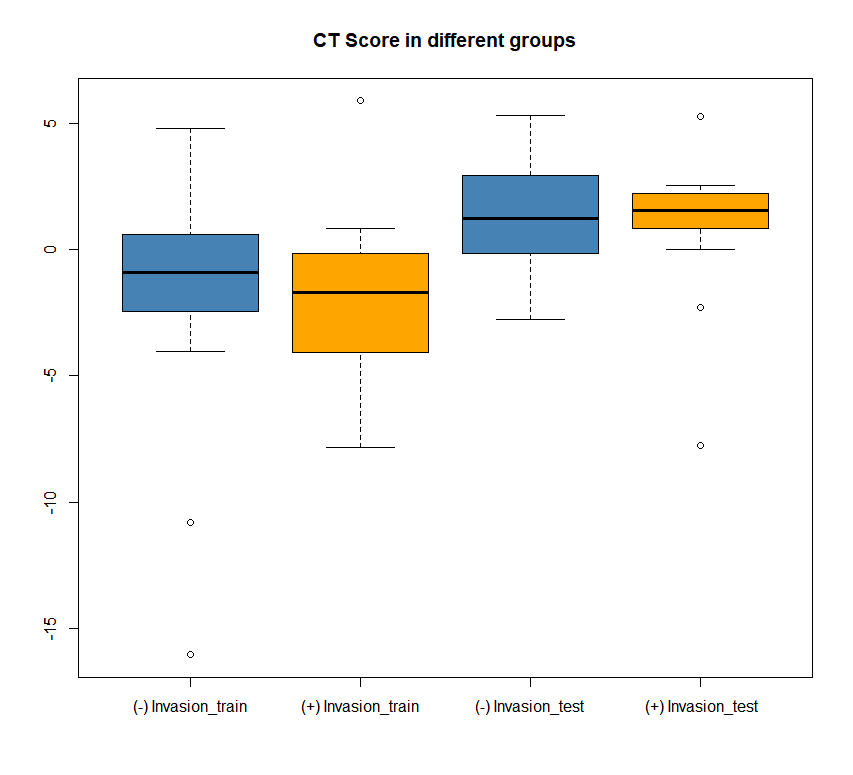

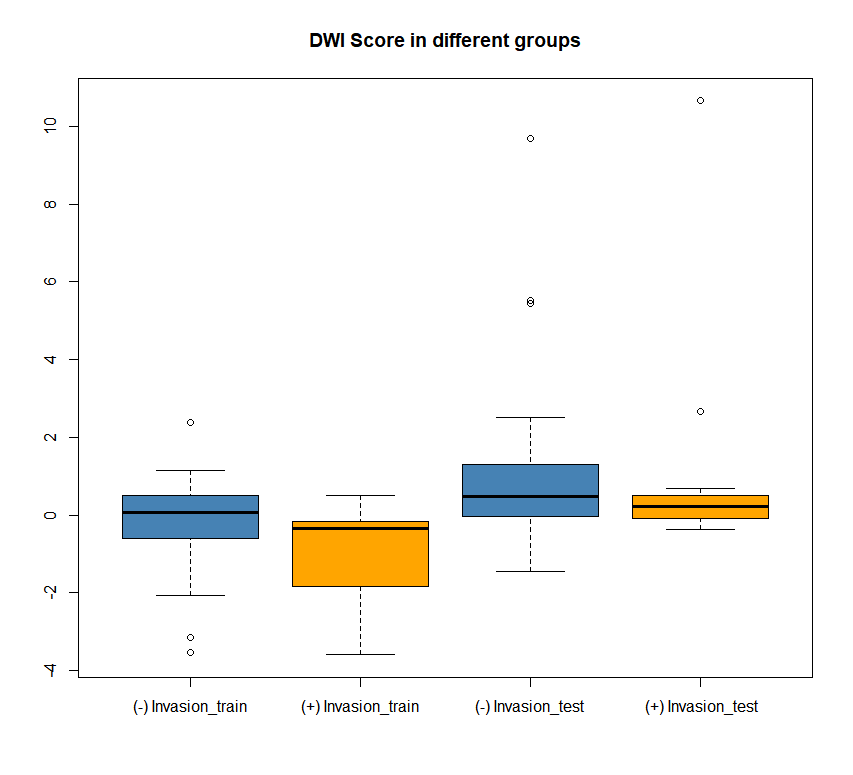

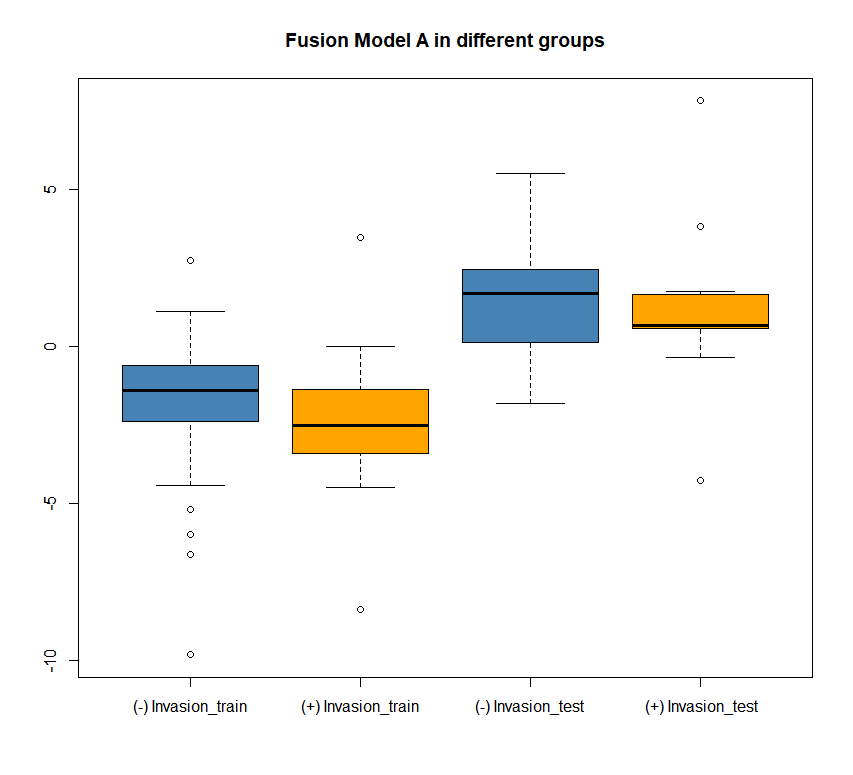

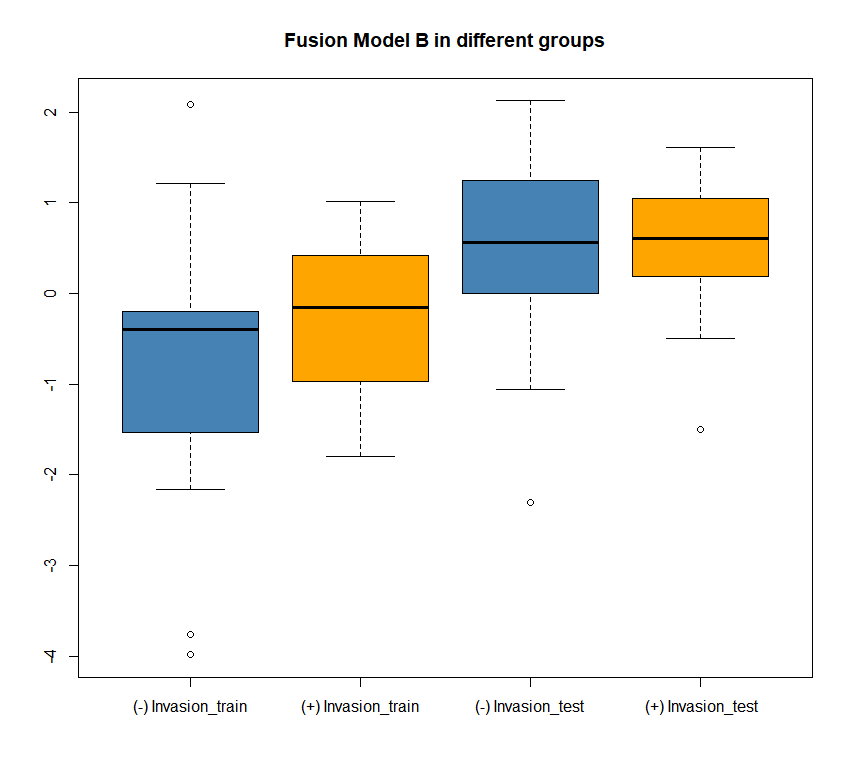


**Supplementary Figure3**. ROC curves of the multimodel radiomics nomogram based on different CT scanners.


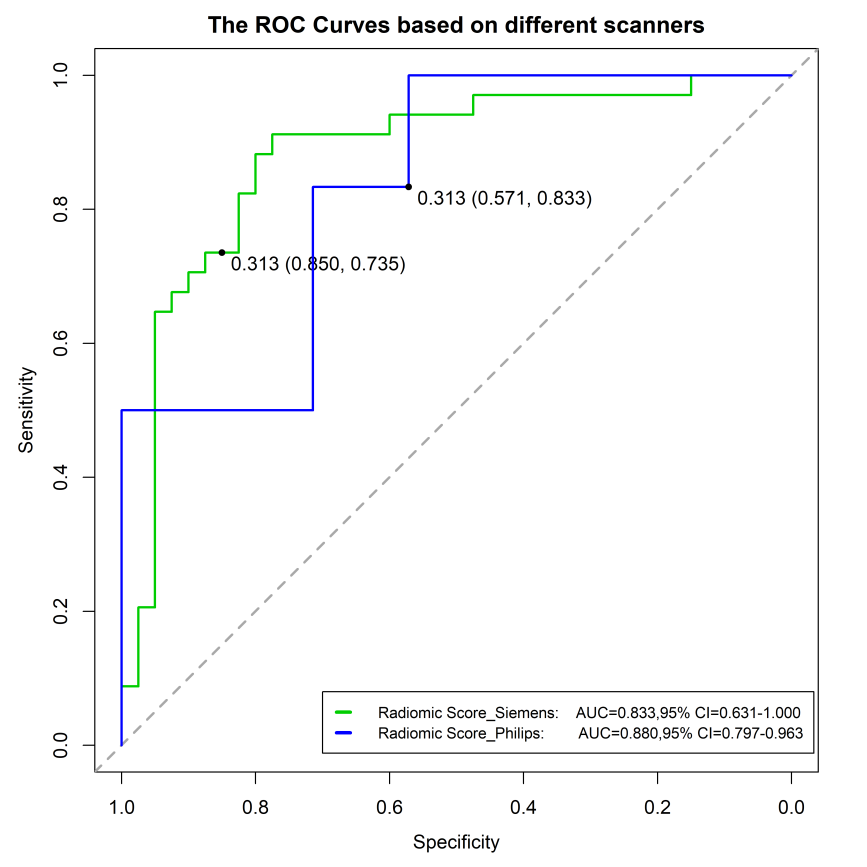


**Supplementary**. Details of features selection based on T2WI, DWI and CT images

( i ) In the process of building T2WI_score, we have used Mann-Whitney U test, after which only 10 features were significant (as follows), and then we used spearman correlation test (here we chose |r|=0.9) to remove redundancy, at last only 6 features (that is T2WI_Feature 1,2,4,5,6,10, as shown in the correlation heatmap), which were less than 10 were remained. Thus, LASSO was not applied.

[1] "HighGreyLevelRunEmphasis_AllDirection_offset4_SD"

[2] "HighGreyLevelRunEmphasis_AllDirection_offset7_SD"

[3] "LongRunEmphasis_angle45_offset4"

[4] "LongRunEmphasis_angle45_offset7"

[5] "ShortRunEmphasis_angle45_offset4"

[6] "ShortRunHighGreyLevelEmphasis_AllDirection_offset7_SD"

[7] "Compactness1"

[8] "Compactness2"

[9] "SphericalDisproportion"

[10] "Sphericity"


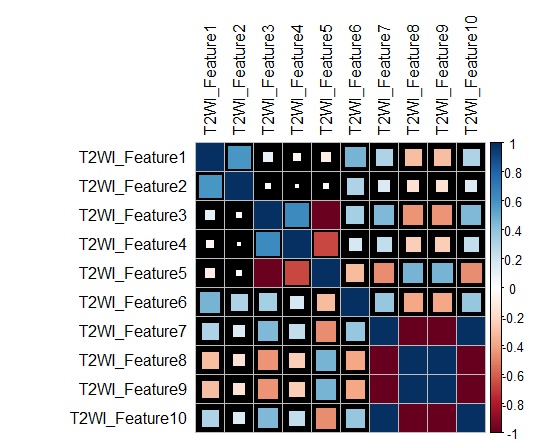


( ii ) In the process of building DWI_Score, LASSO method was used in which lambda was chosen as 0.114, which gave the minimum binominal deviance, and 3 features were remained, as shown in the LASSO figure.

**
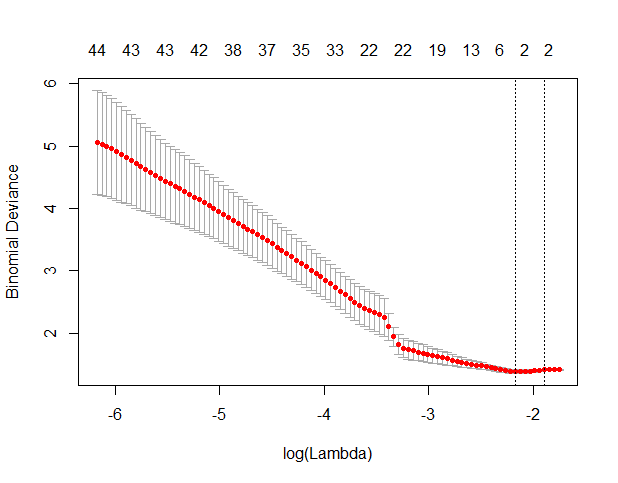
**

( iii ) In the process of building CT_Score, after Mann-Whitney U test and t test, 23 features were remained, as follows. Then LASSO method was used in which lambda was chosen as 0.024, which gave the minimum binominal deviance, and 8 features were remained, as shown in the LASSO figure.

The 23 features left after Mann-Whitney U test and t test:

[1] "ClusterProminence_AllDirection_offset1_SD"

[2] "ClusterProminence_angle90_offset4"

[3] "ClusterShade_AllDirection_offset1_SD"

[4] "ClusterShade_angle135_offset7"

[5] "ClusterShade_angle45_offset7"

[6] "Correlation_angle0_offset7"

[7] "Correlation_angle135_offset7"

[8] "Correlation_angle45_offset7"

[9] "HaralickCorrelation_angle45_offset4"

[10] "HaralickCorrelation_angle45_offset7"

[11] "HaralickCorrelation_angle90_offset7"

[12] "Inertia_AllDirection_offset7"

[13] "Inertia_angle0_offset4"

[14] "Inertia_angle135_offset7"

[15] "Inertia_angle45_offset4"

[16] "Inertia_angle45_offset7"

[17] "InverseDifferenceMoment_angle45_offset7"

[18] "sumAverage"

[19] "ShortRunEmphasis_AllDirection_offset7_SD"

[20] "ShortRunEmphasis_angle90_offset7"

[21] "Maximum3DDiameter"

[22] "IntensityVariability"

[23] "SmallAreaEmphasis"

**
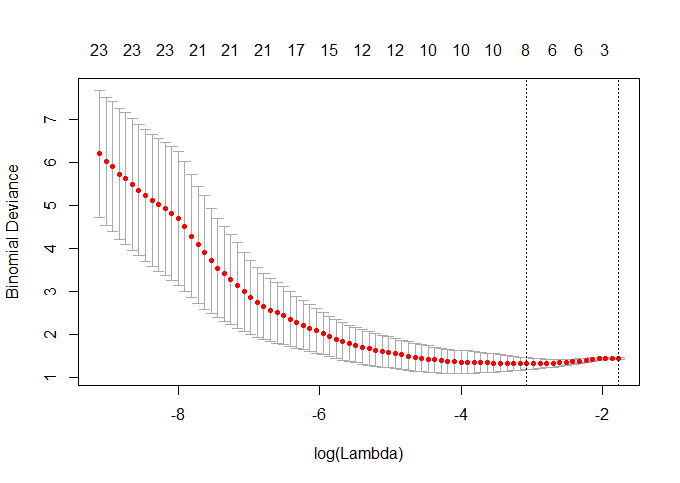
**

**Supplementary.** The single model with two different parameters

The single-mode model was as following:

(1) MR_Score=-0.004+0.855*T2_Score+0.897*DWI_Score.

(2) CT_Score was as the description in the manuscript

The performance compared with a multi-mode model

(i) For the MR_Score: The AUC was 0.812(95% CI: 0.709-0.916) and 0.762(95% CI: 0.579-0.945) in training and validation cohort, respectively.
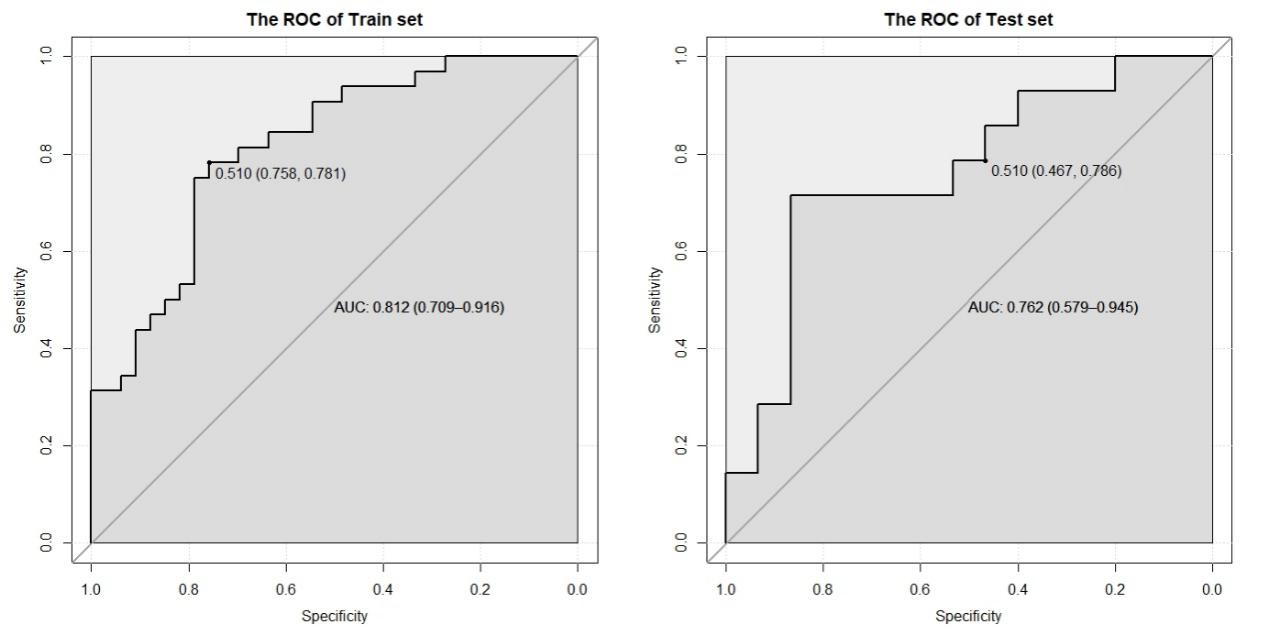


(ii) For the CT_Score: The AUC was 0.804(95% CI: 0.697-0.911) and 0.824(95% CI: 0.643-1.000) in training and validation cohort, respectively.

(iii) For the multi-mode Model A, the AUC was 0.884(95%CI: 0.803-0.964) and0.876 (95% CI: 0.721-1.000) in the training and validation cohort, respectively. The multi-mode Model showed the best performance, although there was no significant differences with other models when using delong test, which may due to the small sample size and the confidence interval was overlapped among the different models. But the sensitivity and specificity were promoted after fusion. The AUC value was not the only one evaluation index. Thus, we choose the multi-mode fusion (that is model A) as the final model after all the comprehensive consideration. Thanks again for your reminder and providing us a new idea.

The specific results are as follows:

Deviance Residuals:

Min 1Q Median 3Q Max

-1.729 -0.924 -0.191 0.956 2.112

Coefficients:

Estimate Std. Error z value Pr(>|z|)

(Intercept) -0.00432 0.29784 -0.01 0.988

T2_SCORE 0.85465 0.36275 2.36 0.018 *

DWI_SCORE 0.89690 0.37432 2.40 0.017 *

---

Signif. codes: 0 ‘***’ 0.001 ‘**’ 0.01 ‘*’ 0.05 ‘.’ 0.1 ‘ ’ 1

(Dispersion parameter for binomial family taken to be 1)

Null deviance: 90.094 on 64 degrees of freedom

Residual deviance: 66.623 on 62 degrees of freedom

AIC: 72.62

> r_spm_step

$CM_Train

Predicted

Actual 0 1

0 25 8

1 7 25

$CM_Test

Predicted

Actual 0 1

0 7 8

1 3 11

$More_information

Train_set Test_set

AUC 0.812 0.762

AUC_95%CI_low 0.709 0.579

AUC_95%CI_up 0.916 0.945

ACC 0.769 0.621

Specificity 0.758 0.467

Sensitivity 0.781 0.786

Threshold 0.510 0.510

Feature num 2.000 2.000

**Supplementary.** The relatively small sample size was a limitation in our study, thus we carefully consulted statisticians and tried to know whether the sample size in validation cohort was sufficient in our study. Shein-Chung Chow [2] had introduced a sample size estimation method for clinical research. The sample size calculation refers to the following formula, in which the two groups (in our study, A was LVI (-), B was LVI (-)) denoted as $A$ and $B$, $\mu$ represents the mean (in our study, mean value was the mean value of radiomic score) in each group. The sample size was calculated respectively:

$$N_{A}=\left( \frac{n_{A}+n_{B}}{n_{B}} \right)\left( \sigma\frac{z_{1-\alpha/2}+z_{1-\beta}}{\mu_{A}-\mu_{B}} \right)^{2}$$

$$N_{B}=\left( \frac{n_{A}+n_{B}}{n_{A}} \right)\left( \sigma\frac{z_{1-\alpha/2}+z_{1-\beta}}{\mu_{A}-\mu_{B}} \right)^{2}$$

where, $n$ is the sample size in the training cohort and $N$ is the sample size for the validation cohort, $\alpha$ is the Type I error, $\beta$ is the Type II error, $1-\beta$ is the power, and $\sigma^{2}$ is the variance of the covariate.

In our study, the sample sizes in the training cohort were $n_{A}=33$ and $n_{B}=32$ with radiomics score (that was based on the fusion model A) means of $\mu_{A}=-1.821$ and $\mu_{B}=1.408$, respectively, and with a standard deviation of $\sigma=2.052$. Therefore, the minimum sample numbers of validation cohorts were 11 in the LVI (-) group and 11 in the LVI (+) group with the desired two-sided significance level of $\alpha$ = 0.05 and power of $1-\beta$ = 95%. In our validation cohort, 15 LVI (-) cases and 14 LVI (+) cases were more than the minimum required sample sizes. However, we didn't have external validation cohort, we would collect more cases that both scanned by MR and CT in our hospital and other hospitals to validate our model in the future. Thanks again for your reminder sincerely. We will improve the robustness and stability in next multi-center study.

1. ：Chow S, Shao J, Wang H. Sample size calculations in clinical research. 2nd Ed. Chapman &Hall//CRC Biostatistics Series 2008.
